# Interplay of circular RNAs in gastric cancer - a systematic review

**DOI:** 10.3389/fsysb.2024.1497510

**Published:** 2024-11-13

**Authors:** Dipanjan Guha, Jit Mondal, Anirban Nandy, Sima Biswas, Angshuman Bagchi

**Affiliations:** ^1^ Bioinformatics Infrastructure Facility Center, University of Kalyani, Kalyani, West Bengal, India; ^2^ Department of Biochemistry and Biophysics, University of Kalyani, Kalyani, West Bengal, India

**Keywords:** circRNA, biogenesis, gastric cancer, early diagnosis, therapeutics

## Abstract

Circular RNAs (circRNAs) have gained prominence as important players in various biological processes such as gastric cancer (GC). Identification of several dysregulated circRNAs may serve as biomarkers for early diagnosis or as novel therapeutic targets. Predictive models can suggest potential new interactions and regulatory roles of circRNAs in GCs. Experimental validations of key interactions are being performed using *in vitro* models, confirming the significance of identified circRNA networks. The aim of this review is to highlight the important circRNAs associated with GC. On top of that an overview of the mechanistic details of the biogenesis and functionalities of the circRNAs are also presented. Furthermore, the potentialities of the circRNAs in the field of new drug discovery are deciphered.

## 1 Introduction

The circular RNAs (circRNAs), a member of the group of RNAs apparently having no-coding potentials, have a profound influence on regulatory pathways and are associated with different types of malignancies, notably gastric cancers (GCs) (Li X. W. et al, 2020). An important feature of such RNAs is their unusual stability due to their covalently closed structure, which allows them to withstand the attacks of the exonucleases (Liu et al., 2022a). The prime activities of the circRNAs include.

• interactions with RNA-binding proteins (RBPs),
• functioning as micro RNA (miRNA) sponges,
 and
• altering gene expression profiles (Zang et al., 2020).

Identifying circRNAs is a bit challenging compared to linear RNAs due to their unique structure. The specific characteristics of circRNAs which set them apart from their linear counterparts are the absence of a capping at the 5′ end and polyadenylated tail at the 3′ end. Instead of traditional markers, specific methods and computational strategies are used to identify and verify circRNAs. Key techniques and markers for identifying circRNAs are as follows:

• Back-splicing junction (Zhang et al., 2016)
• RNaseR resistance (Hansen, 2018)
• Divergent primer PCR (Zhong et al., 2018)
• Northern blot (Schneider et al., 2018)
• Fluorescence *in situ* hybridization (FISH) (Zirkel and Papantonis, 2018)
• Ribosomal RNA depletion (López-Jiménez et al., 2018)

CircRNAs have their abilities to modulate gene expressions and establish their roles in several diseases, like cancer, neurodegenerations, cardiovascular disorders etc. The localization of circRNAs plays a crucial role in their function, and their invasive potential, particularly in the context of cancer. CircRNAs that are localized in the cytoplasm are often associated with invasive properties, especially in cancer. In the cytoplasm, they commonly act as miRNA sponges, which is a mechanism of competitive inhibition (Ma et al., 2018). CircRNAs are also found in exosomes or other extracellular vesicles, where they can be secreted from cells and taken up by neighboring cells or distant tissues. Exosomal circRNAs can also contribute to invasiveness by facilitating long-range communication between cells and thereby promoting metastasis (Di Bella and Taverna, 2024). While circRNAs in the nucleus are typically involved in regulating transcriptional activity or splicing, they are less often associated with invasiveness (Huang et al., 2017). Their unusual stability and tissue-specific expressions make them an ideal choice in personalized medicine (Matsuoka and Yashiro, 2023). As mentioned earlier, the circRNAs have become the focal point of attraction in GC onsets. In GC patients, the circRNAs influence the expressions of specific genes thereby exerting control over tumor growth and metastasis (Li et al., 2019). GC refers to the unwanted growth of malignant cells in the stomach lining or gut tissues. There are several types of GCs, and the most significant among them are adenocarcinoma, gastric lymphoma, gastrointestinal stromal, and carcinoid tumors. This review aims to explain the roles of circRNAs in GC, focusing on tumorigenesis, progression, diagnosis, and therapeutic resistance. The authors further investigate circRNAs’ potential as therapeutic agents and prognostic indicators. The review distinguishes itself by delivering new insights into this field of study by addressing the biotechnological therapeutic potential of circRNAs, analyzing how circRNAs are controlled by drugs or microbes in GC, and providing an updated literature mining on the associated molecular processes.

## 2 Latest trends in circRNA research

Recent researches on circRNAs highlight their significant potential in health maintenance, disease detection, and therapeutic advancements (Fischer and Leung, 2017; Zhou et al., 2022; Haque and Harries, 2017; Verduci et al., 2021).

CircRNAs are also emerging as powerful biomarkers due to their stabilities and specific expression patterns to detect various diseases (Zaiou, 2019). These characteristics make them ideal for non-invasive disease detection methods like liquid biopsies (Wen et al., 2021; Roy et al., 2022). In therapeutic contexts, circRNA-based approaches, including circRNA vaccines, are showing considerable promise. These vaccines may offer enhanced stability and long-lasting effects compared to traditional mRNA vaccines, potentially revolutionizing immunization with fewer doses (Hu et al., 2024; Ren et al., 2024; Wang X. et al, 2022; Li and Xing, 2024). Furthermore, circRNAs have interacting potentials with miRNAs, a process referred to as miRNA sponging, modulating gene expressions (Zang et al., 2020), and influencing therapeutic strategies that may connect to gene therapy research to target specific pathways (TGF-β, β-catenin, MAPK, PI3K/Akt/mTOR signalling pathways) involved in various diseases including GC.

## 3 Biogenesis of circRNAs

### 3.1 Biogenesis of circRNA through intron-pairing-driven circularization forming ecircRNAs

This biogenesis process starts with the transcription of pre-mRNA containing exons and introns. Complementary sequences within flanking introns pair together (Zhao et al., 2022) bringing splice sites close enough to facilitate back-splicing. The event finally leads to the joining of 3′hydroxyl group to a 5′phosphate group present downstream and upstream respectively at the splice site junctions to form a lasso intermediate. This results in exons being joined in a circular form, excising the intervening intronic sequences. The resulting circRNA is covalently closed and does not have a capping at the 5′end and a poly(A) tail at the 3′end forming the exonic circRNAs (ecRNAs/ecircRNAs). This circularization is regulated by RBPs and specialized RNA secondary structures are formed to enhance or inhibit the process (Zhao et al., 2022; Hwang and Kim, 2024; Ebbesen et al., 2017).

### 3.2 Biogenesis of circRNA through RBP-mediated circularization forming exonic-intronic circRNA (EIciRNAs)

Another process of the biogenesis of circRNAs is through RBP-mediated circularization where RBPs bind to specific motifs within the pre-mRNA, often within flanking introns, to stabilize the RNA structure and facilitate the back-splicing process (Hwang and Kim, 2024). These proteins act as scaffolds, promoting the proximity of splice sites, which enhance circularization. Proteins like Quaking can bind intronic sequences to promote circRNA formation by looping out intervening exons and facilitating back-splicing to form EIciRNAs. The resulting circRNA has a covalent linkage between its 5′ and 3′ termini due to absence of the capping and polyadenylation at 5′ and 3′ ends respectively (Conn et al., 2015; Hwang and Kim, 2024; Zang et al., 2020; Chaudhary and Banerjee, 2024).

### 3.3 Biogenesis of circRNA through lariat-driven circularization forming ciRNAs

This mechanism involves the formation of a lariat intermediate during the splicing of pre-mRNA. When classical GU/AG splicing takes place in the pre-mRNAs, exons are skipped, and a lariat-like intermediate containing intron-exon sequences can form (Brown and Simpson, 1998; Zhao et al., 2022). The 5′splice site of an exon joins a branch point within an intron, forming a lariat structure. Back-splicing occurs within this lariat, resulting in a circRNA molecule (Pisignano et al., 2023) named as intronic circRNAs. The excised lariat intron is debranched by enzymes, leaving behind the circRNA. The efficiency of this process is influenced by sequence elements within the introns and exons, as well as cellular factors that regulate splicing (Wilusz, 2018).

## 4 Functions of circRNAs associated with GC

CircRNAs of various kinds have been implicated in the initiation and advancement of GC through their regulation of distinct signalling pathways. These circRNAs typically function through a variety of mechanisms, including the regulation of particular signalling pathways (TGF-β, β-catenin, MAPK, PI3K/Akt/mTOR, Hippo signalling pathways etc.), activation and deactivation of the phosphorylation sites of those proteins, binding with particular proteins that can activate or inhibit those pathways (shown in Table 1), acting as translated proteins, interacting with RBPs, miRNA to affect gene expressions.

**TABLE 1:** Important circRNAs, which are associated with significant pathways are mentioned.

CircRNA	Pathways involved	Targets	Type of circRNA	References
Hsa_circ_0001649	miR-20a/VEGFA Pathway, PI3K/AKT/mTOR Pathway,Wnt/β-Catenin Pathway	miR-20a/VEGFA	Exonic derived host gene	Sun et al. (2020)
Hsa_circ_0015286	STAT3, PI3/AKT pathways	miR-1236-3p	Exonic derived host gene	Su et al., 2019; Xu et al., 2022
circ-LARP4	Hippo signalling pathway	miR-424-5p, LATS1	Exonic derived from LARP gene	Zhang et al. (2017)
circUBE2Q2	Stat3 Pathway	miR-370-3p/STAT3 axis	Exonic derived from UBE2Q2 gene	Yang et al. (2021)
hsa_circ_0000096	PI3K/AKT pathway	miR-145	Exonic derived from host gene	Li et al., 2017; Wang and Dong, 2019
Circ GSPT	PI3K/AKT/mTOR pathway	GSPT1-238aa, Vimentin/BECN1/14-3-3	Exonic derived from GSPT gene	Hu et al., 2024; Zhang et al., 2024
CircAKT3	PI3K/AKT signaling	miR-198, PIK3R1	Exonic derived AKT3 gene	Huang et al. (2019)
CircMTO1	Wnt/β-catenin signaling pathway	miR-199a-3p/PAWR axis, miR-3200-5p/PEBP1 axis, miR-9/p21 axis	Exonic derived from MTO1 gene	Su et al., 2019; Ghafouri-Fard et al., 2021
Hsa_circ_0000520	PI3K/AKT signaling	Unknown	Exonic derived from host gene	Sun et al. (2018)
circPGD	EMT, PI3K/AKT, JNK	miR-16-5p, ABL2	Exonic derived from PGD gene	Garlapati et al., 2021; Liu et al., 2022b
circNRIP1	AKT1/mTOR pathway	miR-149-5p	Exonic derived from NRIP1 gene	Liu et al., 2022a; Yang et al., 2020
circAXIN1	Wnt/β-catenin signalling	AXIN1	Exonic derived from AXIN1 gene	Wang and Dong (2019)
circFGD4	PI3K/AKT signaling	miR-198, PIK3R1	Exonic derived from FGD4 gene	Garlapati et al., 2021; Yang et al., 2020
hsa_circ_0023409	PI3K/AKT pathway	IRS4	Exonic derived from host gene	Garlapati et al., 2021
circ_100876	EMT signaling	miR-665, YAP1	Exonic derived from host gene	Garlapati et al., 2021; Lin et al., 2020
ciRS-7	PTEN/PI3K/AKT signaling	miR-7	Exonic derived from host gene	Garlapati et al., 2021; Liu et al., 2022a
circRBMS3	Tumorigenesis	miR-153, SNAI1	Exonic derived from BMS3 gene	Garlapati et al., 2021; Yang et al., 2020

### 4.1 miRNA sponge

CircRNAs, either competitively or non-competitively, bind to miRNAs, a process referred to as miRNA sponging, and thereby regulating their availability and activity within cells. In contrast to their linear counterparts, circRNAs are resistant to degradation due to their closed-loop structure, making them stable miRNA decoys. In this way the circRNAs prevent them from getting tagged to their target mRNA, leading to the de-repression of miRNA-targeted mRNAs. This regulatory mechanism is crucial in various biological processes (Zang et al., 2020), including development, cellular differentiation and homeostasis (Di Timoteo et al., 2020). Dysregulation of circRNA-mediated miRNA sponging is linked to diseases such as cancer, cardiovascular disorders and neurological disorders, thus highlighting their roles in pathophysiology (Min et al., 2021; van Zonneveld et al., 2021; Shi et al., 2022). Moreover, circRNA-mediated miRNA sponging is one of the prime events that enhances or represses different physiological activities like cell cycle regulation, proliferation, and metastasis (Khanipouyani et al., 2021; Wang Y. et al, 2022). For example, circAKT3 (Huang et al., 2019) interacts with miR-198 to influence the PI3K/AKT pathway in GC. Similarly, circRNA hsa_circ_0023409 sponges miR-542-3p, promoting GC progression through the PI3K/AKT axis. Several other circRNAs, like CDR1as and circ_100876, also regulate the migrations of the GC cells and their corresponding invasion by exerting control over the miRNAs (Lin et al., 2020). YAP1 activation via epithelial-mesenchymal transition (EMT)-related transcriptional pathways are modulated by circRPL15, which sequesters miR-502-3p from OLFM4 mRNA and activates the STAT3 pathway, leading to enhanced GC tissue growth, increased cell motility, invasion and reduced apoptosis (Li Y. et al, 2020). CircRanGAP1 promotes VEGFA expression, angiogenesis, and GC metastasis by inhibiting miR-877-3p (Lu et al., 2020). Upregulation of circRNAs in GC drives proliferation and reduces apoptosis, as shown by various *in vitro* and *in vivo* studies. Repressor circRNAs like circYAP1 inhibit GC progression by exerting control over miR-367-5p thereby modulating the p27 Kip1 expression (Liu et al., 2018). CircOXCT1 interacts with miR-136 to alleviate SMAD4 repression, limiting GC EMT and metastasis (Liu et al., 2020). CircFGD4 expression, reduced in tissues of the GC patients, is connected to a low level of metastasis of the lymph nodes and better prognosis by regulating the miR-532-3p/APC/β-catenin interaction pathway (Dai et al., 2020). Similarly, circREPS2 impedes the metastasis of GC tumours by repressing the RUNX3/β-catenin axis and removing miR-558 (Guo et al., 2020).

### 4.2 Transcriptional regulation by interactions with RBPs

CircRNAs can regulate transcription through interactions with RBPs and chromatin-modifying complexes. CircRNAs may act as scaffolds or decoys for RBPs, altering their availability and, consequently, the transcriptional activity of specific genes. By sequestering these RBPs away from their chromatin targets, circRNAs can alter the accessibility of gene promoters or enhancers, thereby modulating transcriptional activity (Hwang and Kim, 2024). Additionally, circRNAs can interact with RNA polymerase II and transcription factors, affecting their bindings to gene promoters and influencing the initiation or elongation phases of transcription. This interaction can either promote or inhibit the transcription of specific genes, depending on the sequence and structure of the circRNA and its binding partners (Bose and Ain, 2018). Moreover, some circRNAs have been found to localize in the nucleus (Li et al., 2017), where they can directly interact with transcriptional machinery or chromatin to exert regulatory effects. RBP IGF2BP3 (insulin-like growth factor 2 binding protein 3, also called IMP3) performs several post-transcriptional functions. In GC, IGF2BP3 (Yu et al., 2021) acts as an oncogene that stimulates cancer cell proliferation and invasion. One of the most important EMT-related transcription factors SNAIL helps in GC progression. Yu et al. described that circTNPO3 is significantly downregulated in GC. It was revealed from *in vivo* as well *in vitro* analyses that circTNPO3 inhibits the growth and metastasis of GC. In terms of mechanism, circTNPO3 competitively interacts with IGF2BP3 (Yu et al., 2021), which leads to the destruction of the MYC mRNA. This inhibition of MYC and SNAIL which is the target of MYC, is the chief and crucial player in the inducing EMT. CircFNDC3B is another up-regulatory circRNA, which can be seen in GC in cell lines. IGF2BP3, an RBP linked to several malignant tumours, can bind to circFNDC3B. Additionally, IGF2BPs have the potential to stabilize CD44 mRNA by targeting it (Hong et al., 2019). Upregulated circFNDC3B helps in the process of migrating and subsequent invading of GC cells when a tri-complex of circFNDC3B-IGF2BP3-CD44 mRNA is formed (Hong et al., 2019) and the regulation of E-cadherin in GC. Furthermore, the expression of E-cadherin is controlled by the presence of circFNDC3B. This was achieved through a novel mechanism. These encouraging findings offer fresh perspectives on treatment and GC prognosis forecasts.

### 4.3 Translation and translocation potential

CircRNAs can influence protein translation through internal ribosome entry sites (IRES), enabling them to produce proteins independently of the canonical mRNA cap structure (Shi et al., 2020). CircRNAs influence protein translocation by interacting with RBPs like HuR and IGF2BP3, which regulate protein localization and control diseases (Granados-Riveron and Aquino-Jarquin, 2016; Shi et al., 2020; Okholm et al., 2020). Such bindings allow circRNAs to guide or sequester RBPs, potentially impacting subcellular protein localization. CircRNAs may also indirectly influence protein translocation by modulating the expression of miRNAs and RBPs involved in protein trafficking (Wan and Hopper, 2018). In GC, circRNAs like CircMAPK1, which is a derivative of MAPK1, can bind to MEK1 competitively and prevent the phosphorylation event that controls the MAPK signalling pathway.

This circMAPK1 can downregulate GC progression by inhibiting MAPK1 protein synthesis, demonstrating their role in translation regulation (Wang Y. et al, 2022). CircRNAs can produce novel peptides with oncogenic or tumor-suppressive functions, adding complexity to their role. Subcellular localization is crucial for circRNA function, with many circRNAs found in the cytoplasm, influencing post-transcriptional gene regulation, while others are nuclear, participating in transcriptional regulation (Zang et al., 2022). For instance, hsa_circ_0000467 derived from exosomes promotes GC progression by facilitating nuclear translocation of transcription factors or stabilizing mRNA targets (Jiang et al., 2024). CircAGO2, derived from the AGO2 gene, regulates AGO2-miRNA complexes and cancer progression by interacting with HuR protein and thereby reducing the gene silencing pathways mediated by AGO2/miRNA network (Chen et al., 2019). Another circRNA, circTHBS1, associated with poor prognosis in GC, promotes malignant behaviours and EMT through the INHBA/TGF-β pathway by sponging miR-204-5p and stabilizing HuR-mediated INHBA mRNA (Qiu et al., 2022). In terms of mechanism, circTHBS1 sponges the miR-204-5p to increase INHBA expression. The circularRNA helps in stabilizing HuR-mediated INHBA mRNA, which activates the TGF-β pathway and can promote GC.

### 4.4 Protein sponge and direct interaction

**FIGURE 1:**
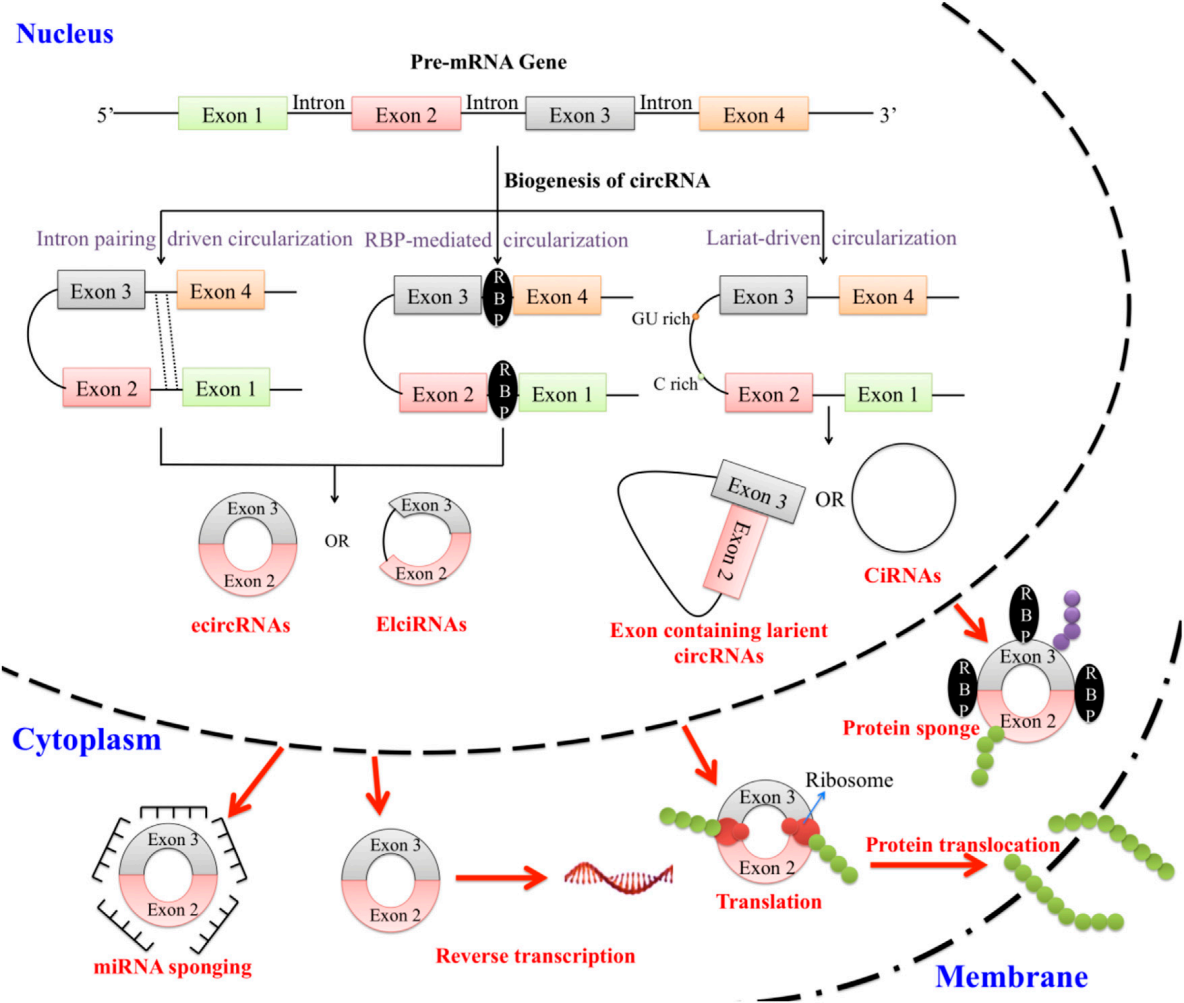
Pre-mRNAs are the parent molecule from which circRNAs are created through reverse splicing. Reverse splicing along with the regular splicing mechanism can exclude the intronic portion to form exonic circRNAs (ecRNAs/ecircRNAs). Few exonic circRNAs have their introns retained and are known as exon-intron circRNAs (EIciRNAs). CircRNAs can also form through intron lariat formation or whole intronic circularization forming intronic circRNAs (ciRNAs). All the pre-mentioned circRNAs are localized inside the nucleus except ecircRNA, which localizes in the cytoplasm. CircRNAs can sponge miRNAs and RBPs. They are also involved in reverse transcription, translation, and protein translocation.

CircRNAs can directly interact with proteins, influencing their stability, localization, and activity. They may serve as sponges for multiple miRNAs or RBPs, forming complex regulatory networks. CircUBE2Q2, an upregulated regulatory circRNA in GC, promotes tumor metastasis by inhibiting the STAT3 pathway through the circUBE2Q2-miR-370-3p-STAT3 axis and exosomal communication (Yang et al., 2021). Similarly, CircURI1, derived from exons 3 and 4 of URI1, is highly expressed in GC in comparison to the wild type tissues (paraGC). CircURI1 inhibits movements and corresponding metastasis of the GC cells both *in vitro* and *in vivo* by interacting with hnRNPM, thereby regulating alternative splicing of migration-related genes and suppressing metastasis (Wang et al., 2020; Wang et al., 2021). Additionally, circMRPS35 impedes the growth and invasion of gastrointestinal cancer cells *in vivo* and *in vitro*. Mechanistically, circMRPS35 acts as a scaffold, recruiting histone acetyltransferase KAT7 to the promoters of FOXO1 and FOXO3a, leading to H4K5 acetylation. This enhances the transcription of FOXO1/3a and their downstream targets, including p21, p27, Twist1, and E-cadherin, thereby inhibiting cell proliferation and invasion. Moreover, a positive correlation exists between circMRPS35 and FOXO1/3a expression in GC tissues (Jie et al., 2020). All these observations demonstrate that circRNAs have pivotal roles in regulating biological processes associated with GC metastasis. The biogenesis and functional mechanism of circRNAs are presented in Figure 1.

## 5 CircRNAs as potential biomarkers for GC

### 5.1 As indicators for diagnosis

Currently, the best biomarker for diagnosing GC early is CA72-4; however, its sensitivity and specificity are not very high. On the other hand, when compared to healthy individuals, circRNAs exhibit unique expression patterns in GC patients, which makes them potentially useful biomarkers for diagnostic purposes of GC while conducting tissue biopsies. Since gastritis is associated with a higher risk of GC, Xie et al. (2018) observed that hsa_circ_0074362 was downregulated in both GC and gastritis tissues, suggesting it could be an early indication of GC (Xie et al., 2018). The levels of hsa_circ_0074362, despite having a poor sensitivity, can be correlated with lymph node metastases and the serum tumor marker CA19-9, suggesting that it may be useful in combination with other markers. A reliable GC biomarker, hsa_circ_0001649 is another circRNA that is downregulated in GC tissues and exhibits great diagnostic accuracy with a sensitivity of 71.1%, and specificity of 81.6% (Li et al., 2017). Moreover, exosomal hsa_circ_0015286 levels in GC patients decrease dramatically after surgery, indicating that it may very well be considered as a tool for the detection and diagnosis of GC in a non-invasive manner exemplifying its capability to act as a potential biomarker (Zheng et al., 2022). Recently, Roy et al. (2022) have discovered a set of eight circRNAs as possible non-invasive indicators for early GC diagnosis.

### 5.2 As biomarkers in prognosis

Patients with GC may have a better prognosis if secondary prevention measures are taken, such as early detection, diagnosis, and treatment. It is now known that circRNAs may be predictive biomarkers. For instance, CircLARP4, which is primarily present in the cytoplasm, sponging miR-424, affects the behaviour of GC cells. According to Zhang et al. (2017) decreased circLARP4 expression in GC tissues is a reliable indicator of GC patients’ overall survival. *In vivo* research revealed that circUBE2Q2 knockdown in conjunction with a STAT3 inhibitor synergistically suppresses the growth of GC, indicating that circUBE2Q2 targeting may improve the efficacy of targeted therapies (Yang et al., 2021). A different serum hsa_circ_0007507 expression levels across GC patients with gastritis, intestinal metaplasia, post-surgery, and recurrence cases suggest that it may be a novel biomarker for GC monitoring and diagnosis (Zhang et al., 2021). Furthermore, a substantial downregulation of hsa_circ_0000096 in GC tissues was reported by Li et al. (2017) have strong diagnostic accuracy.

### 5.3 As therapeutic targets

CircRNAs have been implicated in the development of GC and are important; these findings raise the possibility that circRNAs could be targets for therapeutic intervention. For instance, in GC patients being treated by cisplatin (CDDP), the overexpression of circAKT3 hampers the chances of disease-free survival (DFS) and is independently linked to aggressive tumor characteristics. The expression profile of circAKT3 is found to be higher in the tissues and cells which are resistant to CDDP as compared to their CDDP sensitive counterparts. According to Sun et al. (2018) there is a positive correlation between TNM stage and the expression of CEA in GC plasma, but a negative correlation exists between the concentrations of hsa_circ_0000520 in GC tissues and TNM stage. Hu et al. (2022) found that the novel protein GSPT1-238aa, encoded by circGSPT1, suppresses the growth of GC tumors, suggesting, it may be a special therapeutic target. Circ-MTO1, another circRNA, has been linked to enhanced chemotherapeutic sensitivity, longer DFS, and decreased lymph node metastases in GC (Chang et al., 2022).

Various diagnostic methods have been developed to accurately detect and quantify circRNA expression levels. RNA sequencing (Wu et al., 2022; Szabo and Salzman, 2016) and thereafter different computational tools (Wu et al., 2022; Nguyen et al., 2022; Singh et al., 2022) are needed to detect circRNAs for different purposes.

## 6 Conclusion and future perspectives

**FIGURE 2:**
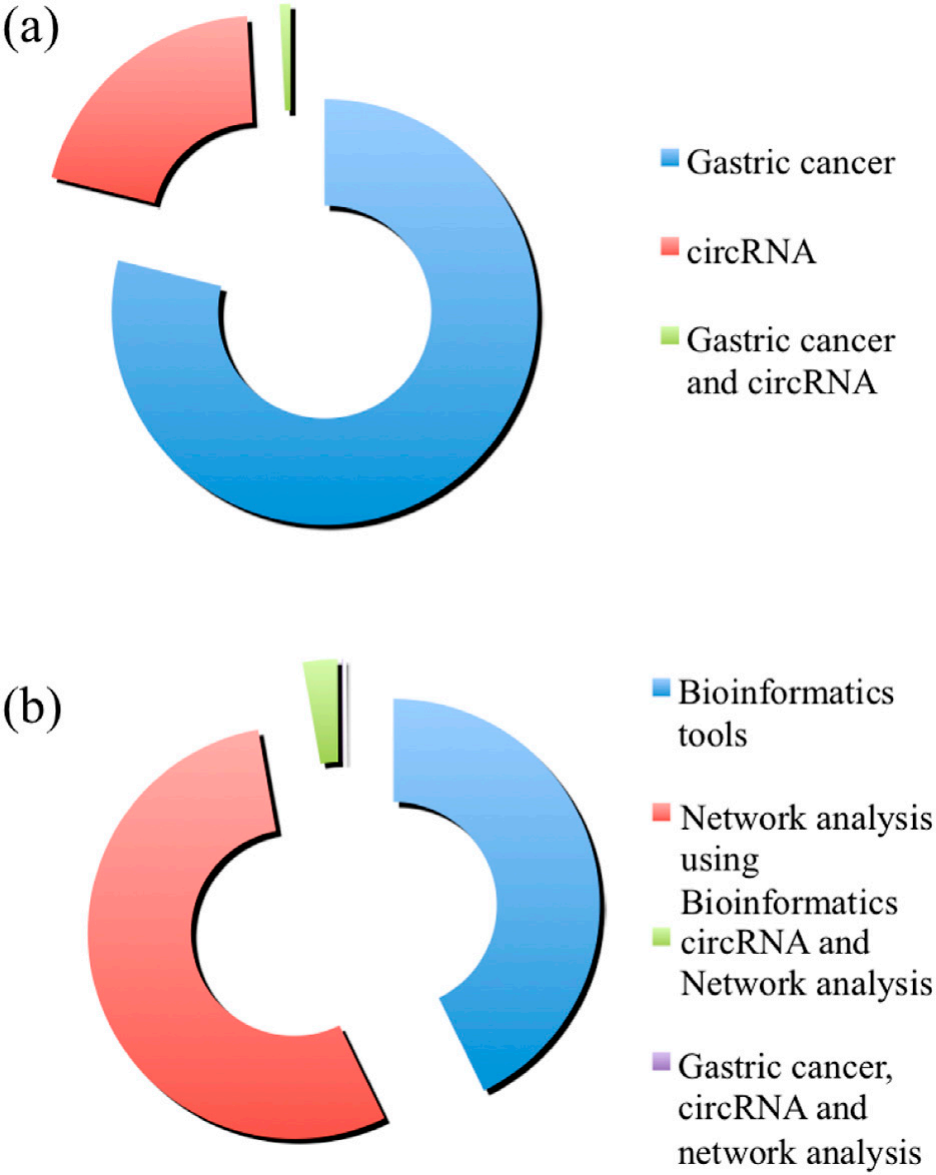
The number of publications in “PubMed” is shown in the chart. Panel **(A)** shows the amount of publications with the keywords “Gastric cancer” (in blue), “circRNA” (in red), and “Gastric cancer and circRNA” (in green). Panel **(B)** shows the amount of publications with the keywords “Bioinformatics tools” (in blue), “Network analysis using Bioinformatics” (in red), “circRNA and Network analysis” (in green), and “Gastric cancer, circRNA and network analysis” (in purple).

Since the last decade, circRNAs have emerged as significant players in the regulation of gene expression, with growing evidence suggesting their critical roles in GC. One of the most cost-effective ways to detect the interplay between circRNAs is through the bioinformatics approach, which is also found to be coming very fast to catalyze this area of research (Figure 2). These stable, covalently closed RNA molecules, circRNAs exhibit unique characteristics, such as high resistance to exonucleases and the ability to act as miRNA sponges, regulators of transcription, and protein scaffolds. In GC, circRNAs have been implicated in key pathological processes, including protein aggregation, and healthy cell death. Researches indicate that circRNAs can influence disease progression by modulating gene expression networks and interacting with disease-related proteins. Future perspectives on circRNAs in GC involve several promising directions. Advanced high-throughput sequencing and bioinformatics tools will facilitate the comprehensive identification and characterization of circRNAs in diseased versus healthy tissues. Functional studies using *in vitro* and *in vivo* models may pave the pathways for the elucidations of the precise functionalities of circRNAs in GC cells and numerous other diseases. Moreover, the development of circRNA-based therapeutics, such as synthetic circRNAs or circRNA inhibitors, holds potential for innovative treatments. Overall, continued research into circRNAs will enhance our understanding of GC and pave the way for novel diagnostic and therapeutic strategies.
